# Coding-complete genomic sequence of bovine viral diarrhea virus isolated from a calf in Taiwan

**DOI:** 10.1128/mra.01218-23

**Published:** 2024-01-30

**Authors:** Chih-Wei Huang, Yu-Ching Chuang, Lu-Jen Ting, Fan Lee

**Affiliations:** 1 Veterinary Research Institute, Ministry of Agriculture, New Taipei City, Taiwan; DOE Joint Genome Institute, Berkeley, California, United States

**Keywords:** bovine viral diarrhea virus, cattle, Taiwan, East Asia

## Abstract

The coding-complete genomic sequence of a Taiwanese bovine viral diarrhea virus (BVDV) was sequenced. Phylogenetic analysis suggested that the Taiwanese isolate belonged to the 1b clade. This study will advance the understanding of BVDV genotypes in Southeast Asia and promote future studies on BVDV epidemiology in Taiwan.

## ANNOUNCEMENT

Bovine viral diarrhea virus (BVDV) is a cattle pathogen causing respiratory disease, enteritis, and immune dysfunction (1). Belonging to the *Flaviviridae* family and *Pestivirus* genus (2), BVDV is a small, spherical, single-stranded, enveloped RNA virus of 40–60 nm in diameter (3). Its genome is a 12.3-kb positive-sense RNA with one open reading frame (ORF) flanked by untranslated regions at the 5′ and 3′ ends (4). The genus *Pestivirus* includes BVDV genotypes (types 1 and 2), classical swine fever virus, and border disease virus (5). Here, we report the coding-complete sequence of a cytopathic BVDV isolate from a calf with bloody diarrhea in Taoyuan, Taiwan.

**Fig 1:**
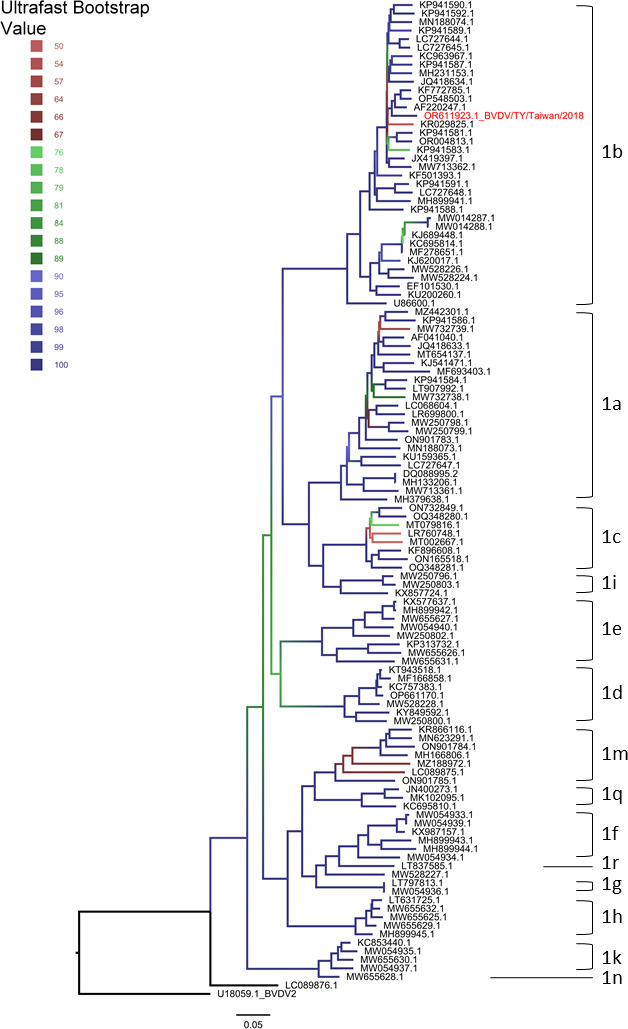
Maximum likelihood phylogeny of bovine viral diarrhea virus genotype 1 based on polyprotein nucleotide sequences. BVDV sequenced in this study is colored in red. The color of the branch indicates the branch support based on 1,000 replicates of ultrafast bootstrap approximation.

A clinical sample was collected from a farm on 27 February 2018. The isolate BVDV/TY/Taiwan/2018, recovered from the lung and liver of a 4-month-old calf with fever, dehydration, and bloody diarrhea, was grown in primary bovine kidney cells following the protocol of World Organisation for Animal Health (6). The cytopathic effects were observed in the secondary passage after 24–48 hours of incubation. Electron microscopy revealed typical pestivirus particles in the cell culture supernatant. Automatic viral RNA extraction was performed using the MagNA Pure Compact Instrument with the MagNA Pure Compact Nucleic Acid Isolation Kit I (Roche, Switzerland) (7). A sequencing library was constructed with Illumina Stranded Total RNA Prep with Ribo-Zero Plus. Sequencing was performed using a 500-cycle (2 × 250 bp paired-end) MiSeq Reagent Kit v2 (Illumina, USA) with a MiSeq sequencer at the Veterinary Research Institute in Taiwan. In total, 6,721,818 reads were generated. Base quality lower than Q30 and PCR-duplicated reads were trimmed using default parameters by BBDuk and Dedupe, respectively, in Geneious Prime 2023 (https://www.geneious.com). A BVDV isolate from China (KF772785) was used as a reference sequence based on the BLAST results of contigs generated using Geneious *de novo* assembler with default parameters. Sequence mapping was conducted by Geneious assembler with low sensitivity setting and 10% maximum mismatches per read. The BVDV genome (OR611923) was 12,238 bp in length (GC%: 45.5%), assembled from 452,509 reads, with an average sequencing coverage of 4,911. The 11,697-bp polyprotein coding region was predicted with the initial codon ATG by ORF finder. Other complete coding sequences of BVDV excluding human-modified isolates were obtained from GenBank. Sequences of the coding region of BVDV were aligned by translated amino acids using MUSCLE (8) implemented in AliView version 1.28 (9). Maximum likelihood phylogeny was reconstructed by IQTREE version 2.2.2.6 (10) using the GTR + F substitution model, selected based on Bayesian information criteria, with 1,000 replicates of ultrafast bootstrap approximation (11) for branch support assessment. The phylogeny was visualized by Figtree version 1.4.4 (http://tree.bio.ed.ac.uk/software/figtree/). The grouping of BVDV was based on previous works (12, 13). The isolate BVDV/TY/Taiwan/2018 belonged to the 1b clade (Fig. 1). Phylogenetically, the most closely related strain of BVDV/TY/Taiwan/2018 was CP7 strain (AF220247) isolated from the USA. We used MEGA X (14) to calculate the nucleotide and amino acid genetic distances of the coding region between the Taiwanese isolate and USA strain, which were 5.57% and 3.34%, respectively.

## Data Availability

Genomic sequence: BVDV/TY/Taiwan/2018 OR611923. Illumina Miseq raw reads: SRR27167855.
